# Searching for Speciation Genes: Molecular Evidence for Selection Associated with Colour Morphotypes in the Caribbean Reef Fish Genus *Hypoplectrus*

**DOI:** 10.1371/journal.pone.0020394

**Published:** 2011-06-08

**Authors:** Ben G. Holt, Isabelle M. Côté, Brent C. Emerson

**Affiliations:** 1 Department of Biology, Center for Macroecology, Evolution and Climate, University of Copenhagen, Copenhagen, Denmark; 2 School of Biological Sciences, University of East Anglia, Norwich, United Kingdom; 3 Department of Biological Sciences, Simon Fraser University, Burnaby, Canada; 4 Island Ecology and Evolution Research Group IPNA-CSIC, La Laguna, Tenerife, Canary Islands, Spain; Lund University, Sweden

## Abstract

Closely related species that show clear phenotypic divergence, but without obvious geographic barriers, can provide opportunities to study how diversification can occur when opportunities for allopatric speciation are limited. We examined genetic divergence in the coral reef fish genus *Hypoplectrus* (family: Serranidae), which comprises of 10–14 morphotypes that are distinguished solely by their distinct colour patterns, but which show little genetic differentiation. Our goal was to detect loci that show clear disequilibrium between morphotypes and across geographical locations. We conducted Amplified Fragment Length Polymorphism molecular analysis to quantify genetic differentiation among, and selection between, morphotypes. Three loci were consistently divergent beyond neutral expectations in repeated pair-wise morphotype comparisons using two different methods. These loci provide the first evidence for genes that may be associated with colour morphotype in the genus *Hypoplectrus*.

## Introduction

Whether speciation can occur in the absence of geographic barriers remains controversial [1]. In this context colour polymorphisms, which occur in a wide range of taxa, provide excellent opportunities for studies of intraspecific evolutionary divergence (e.g. [2]). Many different mechanisms have been implicated in the origin and maintenance of colour polymorphism, including sexual selection, mimicry, predation, crypsis and genetic drift [3]. As all these mechanisms may also contribute to reproductive isolation, colour polymorphisms are often considered as systems potentially undergoing speciation (e.g. [4]).

Marine polymorphisms can provide particularly important insights into general mechanisms of speciation (e.g. [5]). The marine environment has two characteristics which are potentially of major evolutionary importance (e.g. [6]). Firstly, the marine environment is highly expansive, with few clear geographical barriers. Secondly, a highly diverse range of marine taxa produce planktonic larvae, which can facilitate dispersal over long distances. Both factors enable many species to have large distributions, with the potential for strong connectivity between populations, theoretically limiting opportunities for allopatric speciation. To improve our understanding of how species can evolve under such conditions, there is a need to examine closely related groups that show clear phenotypic divergence, attempting to determine how this divergence may have arisen and whether it can lead to full reproductive isolation.

One of the most studied marine radiations, the genus *Hypoplectrus* (the hamlets), provides a useful system for researching the early stages of evolutionary divergence (e.g. [7], [8], [9], [10], [11], [12], [13]). Hamlets (family Serranidae) are small, colourful, predatory fish that are found on coral reefs throughout the Caribbean and tropical western Atlantic. On the basis of colouration, at least thirteen morphotypes can be recognised, 10 of which have been formally described as species. These different forms can often be found sympatrically, with up to seven or eight species occurring on a single reef [8]. The number of species recognised within the genus has been debated, due to the almost complete lack of structural differences among species. This debate has been reinvigorated in light of molecular genetic studies which, using highly polymorphic markers, have revealed little, if any, divergence between colour morphotypes [11], [14], [15]. In contrast, field observations suggest that hamlets have a strong preference for mating with their own colour type, with only approximately 1% of observed spawnings occurring between morphotypes [7], [13]. Hamlets are simultaneous hermaphrodites and their assortative mating behaviour has been linked to ‘egg trading’ between spawning partners [8]. The paradox between molecular results and observations of spawning behaviour has resulted in some confusion, causing many to reconsider the taxonomic status of *Hypoplectrus* morphotypes. Recent studies using nuclear markers, such as microsatellites, have shown very low but significant reproductive isolation between colour morphotypes [11], [12], [13], and it has been suggested that this is an indication of incipient speciation [13]. It is currently unclear whether this genetic isolation between morphotypes is distributed evenly throughout the genome or if, alternatively, gene flow between some areas of the genome are particularly limited, which would suggest that certain genes play an important role in this phenotypic radiation.

In this study we use established outlier detection methods to search for molecular signatures of selection on specific loci between interbreeding populations. Through analysis of Amplified Fragment Length Polymorphisms (AFLPs) we specifically considered whether individual loci are consistently associated with individual morphotypes across different locations. The AFLP technique has a specific advantage over mtDNA and microsatellite markers that have been so far employed for population genetic analysis of *Hypoplectrus* [11], [12]; by efficiently producing hundreds of markers, it has higher potential to detect polymorphism across the genome. Our study took advantage of this feature to search for direct molecular evidence of selection between *Hypoplectrus* morphotypes across different locations. This approach has an advantage over methods such as bulk segregate analysis or QTL mapping in that it can be applied to samples from natural populations and does not require laboratory breeding, which has so far proven to be unfeasible for these fish [7].

## Materials and Methods

### Sampling

**Figure 1:**
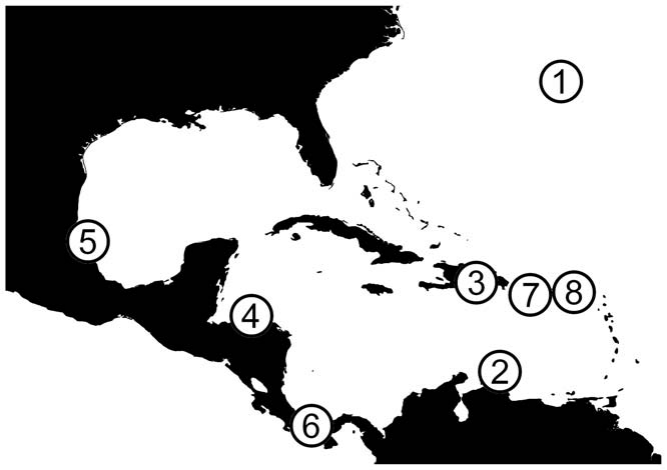
Map of sampling locations for AFLP analysis of *Hypoplectrus* morphotypes. 1 = Bermuda, 2 = Curaçao, 3 = Dominican Republic, 4 = Honduras, 5 = Mexico, 6 = Panama, 7 = Puerto Rico, 8 = U.S. Virgin Islands.

Samples were collected using SCUBA, from coral reef sites at eight sampling locations (i.e. countries) distributed across the Caribbean basin (Fig. 1). We refer to all individuals of a particular colour form at a particular location as a ‘morphotype population’ and all individuals of a particular colour form from throughout the *Hypoplectrus* distribution as a ‘global morphotype population’. Fish were sampled by using micro-spears or “hook and line” whereby baited, barbless hooks were offered to fish. Hooked fish were released after fin clipping. Fin clips were removed from the dorsal and anal fins and placed in ethanol and stored at 4°C. The morphotypes sampled and number of individuals collected varied among locations, depending on local abundance (Table 1).

**Table 1:** *Hypoplectrus* samples collected for AFLP analysis.

	*H. chlorurus*	*H. nigricans*	*H. puella*	*H. unicolor*	V white	Total
Bermuda			90			**90**
Curaçao	42		11	32		**85**
Dominican Republic	4	24	31	33		**92**
Honduras		4	39			**43**
Mexico		34			44	**78**
Panama		25	17	4		**46**
Puerto Rico	38	6	9	6		**59**
U.S. Virgin Islands	4		31			**35**
**Total**	**88**	**93**	**228**	**75**	**44**	**528**

Columns represent described species (with the exception of V white, which refers to the Veracruz white morphotype, endemic to this part of the Gulf of Mexico), rows represent sampling locations.

### AFLP procedure

The AFLP procedure was based on Vos *et al.*, [16]. DNA was isolated from ≈0.5 cm^2^ sub-samples of fin clips, using a phenol-chloroform extraction method [17]. Extractions were checked for concentration and purity to ensure isolated DNA was suitable for the production of clear repeatable AFLP profiles. For each sample, 100 ng of DNA (10 µl solution) was added to 1.4 µl restriction digestion mixture containing 1 U EcoRI and 1 U MseI restriction enzymes, along with 1.1 µl 10×TA reaction buffer (100 mM Tris-Ac pH 7.9, 100 mM MgAc, 500 mM Kac, 10 mM dithiothreitol (DTT)) and 3 µg BSA. DNA was digested for three hours at 37°C. Digested DNA was added to 5.5 µl of ligation mastermix containing 1 µl 5× T4 ligase buffer, 0.5 U T4 ligase, 25 pmol of EcoRI Adaptor and 25 pmol of Mse Adaptor (Table S1). This solution was incubated overnight at 16°C and gel electrophoresis used to ensure that the DNA was fully digested. 2 µl of diluted digestion mixture was used for the pre-selective PCR, which was run in 10 µl total solution containing 5 pmol of primer Eco P and 5 pmol of Mse P (Table S1), along with 1 µl 10× PCR buffer, 15 nmol MgCl_2_, 2 nmol dNTP and 0.25 U Taq DNA polymerase. The pre-selective PCR amplification comprised an initial denaturation step of two minutes at 94°C, followed by 20 cycles of denaturation at 94°C for 20 seconds, annealing at 56°C for 30 seconds and final extension at 72°C for two minutes.

Pre-selective amplifications were checked using gel electrophoresis and then diluted to 1∶20 concentration by the addition of sterile distilled water. 1 µl of pre-selective PCR amplification was used as the template for the selective PCR, which was added to 9 µl PCR mixture containing 5 pmol of each selective primer (Table S1), 1 µl 10× PCR buffer, 20 nmol MgCl_2_, 2 nmol dNTP and 0.25 U Taq DNA polymerase. Eco P selective primers were labelled with either FAM or NED fluorescent labels for genotyping. PCR products were diluted 10× with sterile distilled water, and 5 µl of diluted product of FAM-labelled samples were mixed with an equal quantity of NED-labelled samples for multiplex genotyping. 0.5 µl of the combined diluted product was then added to 9.45 µl HiDi formamide and 0.05 µl ABI ROX size standard. Prior to genotyping all samples were denatured at 95°C for three minutes and then quenched on ice for a further three minutes. Control samples were added to every 96-well PCR reaction plate to ensure repeatability between runs.

Samples were genotyped using an ABI 3730 capillary DNA analyser. The fluorescent spectra of NED and FAM labels do not overlap; however, the possibility that the two combined samples interfered with each other's AFLP profiles was investigated by genotyping several individuals, both singly and also after combining with an alternatively labelled sample. Combined dye samples proved to be equally as reliable as single dye samples. Primer combinations were chosen after screening 32 different primer combinations for profile quality (i.e. whether the presence or absence of individual peaks could be clearly determined) and polymorphism (i.e. there appeared to be high variation in peak presence/absence between individual profiles). Primer pairs showing the highest levels of polymorphism were selected for further analysis.

### AFLP scoring

AFLP profiles were initially viewed using the ABI Genemapper v3.0 software and sets of ‘bins’ were created for each primer combination to record the presence or absence of AFLP peaks for each individual, for each primer combination. This software also provided an additional check for the interference between the two dyes contained in our final samples, as it highlights any peak that may cause or result from this problem. All highlighted peaks were checked for “bleed through” (i.e., the creation of a false peak of one colour due to extremely high intensity of a peak of the other colour) but this never occurred. The peak heights (in relative fluorescence units, RFU) for all individuals were then exported into the R statistical package for scoring using the R script AFLPscore v1.3 [18]. We extracted the DNA of 20 individuals (including individuals of several morphotypes and from several locations) twice and separate AFLP profiles were produced for these extractions for every primer combination. These “double extractions” were then used to optimise AFLPscore threshold scoring values and to determine the repeatability rates for the AFLP markers (i.e. the % of markers that are consistently scored between extractions from the same individual). The thresholds that gave the lowest error rate for each of our primer combinations were used in the final genotyping of all individuals.

### AFLP analysis

Mean *F_ST_* values for pair-wise comparisons of hamlet morphotype populations were calculated using the software AFLPsurv [19]. Significance values were calculated using 1000 permutations and represent the probability of finding a value as high as or higher than the observed value by chance. Any locus linked to specific *Hypoplectrus* morphotypes will be expected to exhibit relatively high frequency disequilibrium across morphotype samples, and therefore higher *F_ST_* values. AFLP data were analysed for signs of diversifying selection between morphotypes using two different methods, in order to check for consistency across methodologies. Firstly, data were analysed using Mark Beaumont's Dfdist program (available at http://www.rubic.rdg.ac.uk/~mab/), which uses the method described in Beaumont & Nichols [20]. Dfdist uses computer simulations to model AFLP loci under neutral conditions, given a set of user-defined evolutionary parameters. Loci with high *F_ST_* values might be considered as being under selection; however, as locus *F_ST_* values are expected to vary according to the degree of heterozygosity of the loci concerned, Dfdist also produces *F_ST_* values for simulated AFLP loci. *F_ST_* values were calculated using the Bayesian method developed by Zhivotovsky [21]. Initially, *F_ST_* values were calculated for all observed AFLP loci that were polymorphic in either of the two morphotype populations considered in a pair-wise comparison. Mean *F_ST_* values for neutral loci were then estimated by removing the highest and lowest 30% of *F_ST_* values and subsequently calculating the mean *F_ST_* value of the remaining loci to give the “trimmed” mean *F_ST_*, as suggested by the software developers. Dfdist parameters were then set to return a simulated mean *F_ST_* value that closely matched, but always exceeded, the empirical mean trimmed *F*st value. 50,000 simulated loci were produced for each empirical pair of morphotype populations considered, with simulated sample sizes set to match those of the actual samples. Initial tests suggested that the distribution of simulated loci was robust to changes in effective population size (*N_e_*) and mutation rate (*μ*), and these parameters were set to 10,000 and 1×10^−5^ respectively, with these values being considered conservative for the study system. Simulated loci were then used to generate 95% and 99% quantile distributions for neutral loci and to generate *P* values representing the probability that the observed *F_ST_* value for an empirical locus could be matched or exceeded under neutral conditions. Empirical loci with *F_ST_* values above simulated quantiles were designated as 95% and 99% outliers accordingly. Morphotype populations with very small sample sizes (i.e., six or fewer individuals) proved to have insufficient statistical power and were excluded from this analysis. *P* value distributions for all empirical loci were used to estimate how accurately the simulated dataset represents the empirical dataset. An accurate simulation would be expected to return approximately the same number of *P* values that are above and below 0.5 (M. Beaumont, pers. comm.). Therefore the number of *P* values above 0.5 was subtracted from the number of *P* values below 0.5 for each pair-wise morphotype population comparison, and the mean of these differences compared to zero using a one-sample *t* test.

The frequencies of 95% and 99% outliers detected using Dfdist were checked for the assumptions of parametric analysis and then compared with relevant neutral expectations. Neutral expectations were assumed to be 5% and 1% of the total number of polymorphic loci for each comparison, for the 95% and 99% levels respectively. The pair-wise comparisons included in this analysis were all nine sympatric morphotype comparisons (excluding morphotype populations with sample sizes of six or fewer individuals) as well as nine randomly selected allopatric same-morphotype comparisons and nine randomly selected allopatric different-morphotype comparisons. The mean % of outlier loci for each type of morphotype population comparison was compared against neutral expectations.

Loci shown to be outliers in comparisons between the same two morphotypes in more than one location are more likely to be genuinely subject to selection between morphotypes, rather than the result of selection that is solely present in one location, or type I errors. Pairs of morphotypes that provided sufficient sample sizes at multiple locations were analysed for the presence of such loci. Any loci that were shown to be above the 99% significance level, simulated by Dfdist, in all available comparisons of the same two sympatric morphotypes (i.e., in more than one location) were designated as “99Sel” and those which were shown to be above the 95% significance level were designated as “95Sel”. To consider the possibility of loci that may be under selection between locations, loci that were significant outliers in same-morphotype allopatric comparisons (but not in sympatric different-morphotype comparisons) were designated as “99Geo” and “95Geo”, for loci consistently above the 99% and 95% significance level respectively. The number of these categories of loci expected to be found under neutral conditions was also calculated using the formula in Nosil *et al.* [22], adapted in our case to include the number of polymorphic loci in this study and the probabilities appropriate for our study design (i.e. 0.05 for 95% significance levels and 0.01 for 99% significance levels). The number of repeated outliers found at each significance level was then compared with neutral expectations using exact binomial tests. Allopatric different-morphotype comparisons were not considered in the repeated outlier analyses as they are not likely to be independent from the other comparisons.

The second analytical method we applied to our data was a fully Bayesian approach developed by Foll & Gaggiotti [23], which uses the software Bayescan (available at http://www-leca.ujf-grenoble.fr/logiciels.htm). This method extends the approach developed by Beaumont & Balding [24], in order to allow the use of dominant markers (such as AFLPs) and to rigorously estimate the probability that each specific locus is subject to selection, rather than solely returning the probability of a locus being selectively neutral. To provide an independent test of the repeated outliers detected using Dfdist, the same pair-wise comparisons were analysed using Bayescan and loci showing probability values higher than 0.7 for the likelihood of being under directional selection were noted (as per [23]). Repeated Dfdist outliers that the Bayescan did not find to be under selection in either comparison are considered to be false positives, those which are found to be under selection in only one comparison may be false positives or, alternatively, may represent local selection that is not consistent across morphotype populations. Outliers that are shown in both pair-wise comparisons using both methods are likely to be under selection between morphotypes.

## Results

### Samples

A total of 528 hamlets were sampled from eight different locations representing five different colour morphotypes, with considerable variation among the numbers of each morphotype found at each location (Table 1). Representative images of the morphotypes sampled at each location are presented in Figure S1. A total of 436 scorable AFLP loci were produced with an overall repeatability rate of 97.1% (see Table S1 for primer combinations). Of these, 423 loci were polymorphic, i.e., they were scored differently in at least one individual.

### Divergence among morphotypes and locations

The overall mean *F_ST_* values among morphotypes across the region and among sampling locations were similar: 0.051 and 0.052, respectively (for pair-wise comparisons, see Table 2). These values will be influenced by regional variation in the numbers of morphotypes sampled. The mean *F_ST_* between sympatric morphotypes was 0.058, with all pair-wise comparisons being significantly greater than zero, with the exception of the comparison of two morphotypes from Honduras. Within morphotypes, geographical population structure had a mean *F_ST_* value of 0.060; however, there was large variation between morphotypes, ranging from 0.020 among *H. chlorurus* populations to 0.114 among *H. nigricans* populations. *F_ST_* values for all sympatric inter-morphtype comparisons and all allopatric intra-morphotype comparisons are presented in Tables S2, S3, S4, S5 and S6.

**Table 2:** Mean *F_ST_* values from AFLP data for pair-wise comparisons of *Hypoplectrus* global morphotypes (i.e. all individuals of a given morph collected across all locations).

	*H. chlorurus*	*H. nigricans*	*H. puella*	*H. unicolor*	Veracruz white
*H. chlorurus*	-	**0.090**	**0.044**	**0.053**	**0.057**
*H. nigricans*		-	**0.046**	**0.041**	**0.056**
*H. puella*			-	**0.055**	**0.031**
*H. unicolor*				-	**0.039**

All values are significantly higher than zero at the 0.01 level.

### Outlier frequency analysis

**Figure 2:**
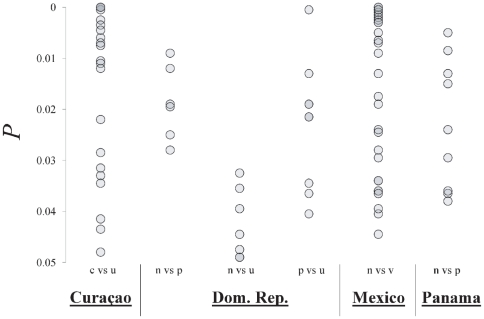
*P* values for outlier loci produced by comparisons of sympatric *Hypoplectrus* morphotype AFLPs. Only comparisons with suitable sample sizes are shown. See table S7 for loci and outlier numbers for all comparisons. Abbreviations represent the morphotypes included in each comparison: c = *H. chlorurus*, n = *H. nigricans*, p = *H. puella*, u = *H. unicolor*, v = Veracruz white.

The simulated *P* values for the empirical loci generated a mean of 8.3 more loci above 0.5 than below 0.5, which is significantly greater than zero (*t*_38_ = 2.16, *P* = 0.037); hence, our simulated values may be conservatively high. However, this imbalance is not large given the number of loci considered and the slightly conservative nature of the simulation helps to protect against false positives. Outlier data for some comparisons were not normally distributed (Kolmogorov-Smirnov tests, *P*<0.05) and therefore all values were compared with neutral expectations using non-parametric Mann-Whitney tests. Overall, there were significantly more outliers at the 99% level than would be expected under neutral conditions, but the number of 95% outliers did not differ significantly from neutral expectations (Table 3). Comparisons of sympatric morphotypes produced outlier numbers that were consistent with neutral expectations. *P* values for the outliers produced by sympatric comparisons are shown in Fig. 2. Randomly selected allopatric, same-morph comparisons showed significantly more outliers than expected at the 99% level but not at the 95% level. Randomly selected comparisons involving different morphs at different locations produced the highest frequency of outliers, which were significantly higher than expectations at both quantile levels. The results for all comparisons selected in this analysis are presented as Table S7.

**Table 3:** Frequencies of outliers found in pair-wise comparisons of *Hypoplectrus* morphotype populations (i.e. individuals of a given morph at a given sampling location).

Comparison	Observed mean %	Expected mean %	Mann Whitney U	*P*
sd95	5.31	5	36	0.671
sd99	1.99	1	36	0.671
as95	6.39	5	36	0.671
as99	2.27	1	0	<0.001
ad95	6.92	5	18	0.034
ad99	2.61	1	18	0.034
all95	5.05	5	36	0.671
all99	2.20	1	0	<0.001

sd = sympatric and different morphotypes, as = allopatric and same morphotypes, ad = allopatric and different morphotypes with nine randomly selected comparisons of each type, all = all comparisons combined. 95 = outliers with *F_ST_* values above the 95% simulated quantile, 99 = outliers with *F*st values above the 99% simulated quantile. ‘Expected mean’ represents the mean percentage of outliers expected. Mann Whitney U and *P* represent the non-parametric test statistics and statistical significance of the comparisons between observed and expected (simulated) results.

### Repeated outlier analysis

**Figure 3:**
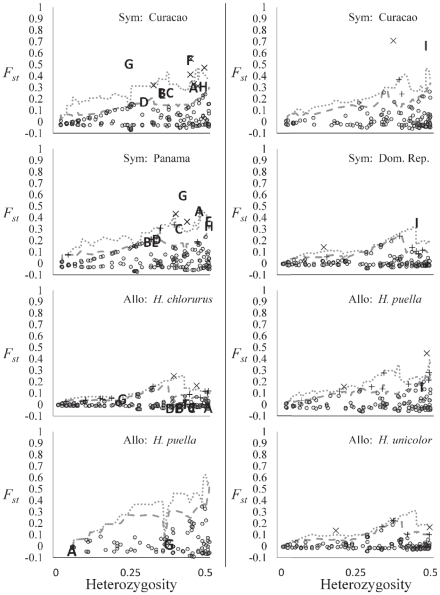
*F_ST_* values for AFLP loci used for repeated outlier loci detection. Left hand plots represent pairwise comparisons for populations of *H. chlorurus* and *H. puella* in Curacao and Panama. Right hand plots represent pairwise comparisons for populations of *H. puella* and *H. unicolor* in Curacao and Panama. “Sym” refers to sympatric comparisons of the two morphotypes concerned (followed by the location), “allo” refers to allopatric comparisons of the two locations concerned (followed by the morphotype). Letters refer to loci repeated across sympatric comparisons (see Table 4 for full loci names and further details). Dashed lines represent the simulated 95% *F_ST_* level, dotted lines represent the simulated 99% *F_ST_* level. Repeated outlier comparisons that did not return any outliers repeated across sympatric comparisons are not shown.

*Hypoplectrus chlorurus*, *H. nigricans*, *H. puella* and *H. unicolor* each provided suitable sample sizes for repeated outlier analysis. A total of 10 loci were identified using the Dfdist method as being potentially under selection between morphotypes at either the 99% or the 95% level (Fig. 3, Table 4). The frequency of repeated outliers showed the opposite pattern to that shown by general outlier frequencies, with sympatric morphotype population comparisons producing more repeated outlier loci than allopatric same morphotype comparisons. The numbers of 95Sel and 99Sel loci were significantly higher than expected under neutral conditions (both *P*<0.001). The numbers of 95Geo and 99Geo loci did not significantly differ from null expectations, however, the relatively small sample sizes for *H. puella* in these analyses is likely to have reduced the probability of detecting loci in disequilibrium in these comparisons involving this species.

**Table 4:** *Hypoplectrus* AFLP loci with *F_ST_* values that were significantly higher than expected in the absence of selection between colour morphotypes, in pair-wise comparisons at two separate locations, using both Dfdist and Bayescan outlier detection methods.

	Code	Dfdist		Bayescan
*Locus*		*95Sel*	*99Sel*	*P>0.7*
Eaga/Mcac121	A	c vs p	c vs p	One (0.66 & 0.95)
Eaga/Mcac168	B	c vs p		One (0.75 & 0.65)
Eaga/Mctc309	C	c vs p		Both (0.74 & 0.80)
Eatc/Mcac206	D	c vs p		None
Eatc/Mcac365	E	c vs p		None
Eatc/Mcag262	F	c vs p		One (0.70 & 0.58)
Eatc/Mcag330	G	c vs p	c vs p	Both (0.74 & 0.77)
Eatc/Mcag77	H	c vs p		None
Eatc/Mctt136	I	n vs p, p vs u	p vs u	n vs p (none) & p vs u (one, >0.99 & 0.65)
Eatc/Mctt138	J	c vs p	c vs p	Both (0.93 & 0.79)

Sel95 and Sel99 = loci that are above the 95% and 99% quantiles simulated by Dfdist. Abbreviations represent the morphotype comparison that yielded the repeated outlier: c = *H. chlorurus*, n = *H. nigricans*, p = *H. puella*, u = *H. unicolor*. Bayescan results show the number of pair-wise comparisons yielding support for each outlying locus. Values in parentheses represent the exact *P* values for each geographical comparison in alphabetical order of locations (not shown if no support exists). Codes given apply to Figure 3. See text and Table 1 for location details.

The Bayescan analysis showed mixed support for the repeated outliers identified using Dfdist. Three of the outliers repeated across different comparisons of *H. chlorurus* and *H. puella* were also identified as being repeatedly under selection using Bayescan. The remaining seven Dfdist outliers were either only identified in one comparison (four outliers) or not identified in any comparison (three outliers) as being under selection between morphotypes by this analysis (Table 4).

## Discussion

Using a genome-wide scanning approach, we found evidence for consistent selection occurring between pairs of *H. chlorurus* and *H. puella*, and between *H. puella* and *H. unicolor* pairs, despite observing low to moderate overall divergence among colour forms. Three AFLP loci were identified as being under selection between morphotypes in pair-wise comparisons of sympatric morphotypes at two different locations, using both the Dfdist and Bayescan methods. These loci represent the first evidence for selection between morphotypes influencing the *Hypoplectrus* genome across sampling locations.

The repeated outlier loci identified are likely to be linked, either physically or otherwise, to genes coding for *Hypoplectrus* colour pattern. An AFLP outlier analysis, similar to ours, on intertidal snails [25] was followed up by sequencing of both outlier and non-outlier loci using bacterial artificial chromosome libraries [26], allowing speculation regarding the phenotypic effects of outlier loci. Applying these techniques to the repeated outliers uncovered in this study could result in a fundamental step forward in understanding exactly how *Hypoplectrus* morphotypes differ from each other, potentially identifying regions of the genome under selection. Comparative analysis of divergent and non-divergent loci may consequently prove to be informative regarding the history of polymorphism in this genus.

The significant repeated outliers were detected above consistently low, but significant, genetic divergence between pairs of morphotypes (Table 2). Our *F_ST_* values, derived from the most comprehensive taxonomic and geographic sampling to date, are in agreement with previous analyses using AFLPs [15] and microsatellites [11], [12]. These results suggest that *Hypoplectrus* morphotypes do represent more than simple colour variants of a single species and that selection acting on a limited number of loci is indirectly resulting in low level significant isolation in other parts of the *Hypoplectrus* genome.

For the majority of loci, differentiation between sympatric morphotypes appears to be no more consistent than differentiation shown between allopatric populations of the same morphotype (Table 3). Overall, pair-wise comparisons of sympatric morphotype populations produced more outliers than expected under neutral conditions but this difference was not significant. By comparison, analysis of allopatric populations composed of the same two morphotypes did reveal a significantly higher number of 99% outliers than expected under neutral conditions, although the number of 95% outliers did not significantly exceed null expectations.

The relatively high level of genetic isolation demonstrated for the two sympatric morphotypes sampled in Mexico is surprising, as there was no *a priori* reason to suspect this comparison to differ from the others. This comparison produced an *F_ST_* of 0.212, far larger than the level of isolation measured in all of other pair-wise comparisons (all other *F_ST_*<0.09). Other unusual features of this region include the fact that just two morphotypes are abundant (BGH, personal observations) and one is an endemic morphotype, the Veracruz white. The only other study to consider hamlets from the Gulf of Mexico [10] found that Veracruz whites tend to feed at a higher trophic level than the sympatric *H. nigricans* population. This species pair may be an interesting focus for future research to determine what has driven the divergence between the two morphotypes in Veracruz.

### Implications for the Hypoplectrus species complex

The hamlet system has attracted considerable attention as a potential case study for speciation in the marine environment. However, the identification of loci specifically associated with certain morphotypes does not necessarily imply that divergence between morphotypes is currently ongoing. An alternative explanation for these results is that assortative mating, linked to the simultaneous hermaphroditism in these fish [8], is maintaining divergence between morphotypes but inter-morphotype reproductive isolation is not fully complete and some successful interbreeding between morphotypes is limiting further divergence. Gene flow between morphotypes would also explain the lack of association between mitochondrial sequence haplotypes and morphotypes shown in other studies [11], [14], [27].

Substantial inter-morphotype gene flow may at first seem to contradict the results of extensive spawning observations, in which only ∼1% of matings occurred between different morphotypes [7], [13]. However, even this level of interbreeding could be consistent with our observed *F_ST_* values given the likely size of the majority of *Hypoplectrus* morphotype global populations. The possibility of infrequent hybridization preventing speciation has been suggested for other systems [28], [29], [30], whereby equilibrium between convergence and divergence is maintained by occasional gene flow.

### Conclusions

Our results show evidence for consistent selection between at least two *Hypoplectrus* morphotypes (*H. chlorurus* and *H. puella*) and highlight three AFLP loci that show good evidence for being directly affected. Selection between colour forms appears to play a role in maintaining this complex of morphotypes; however, this has not resulted in consistent divergence for the vast majority of the loci studied. The low but significant genetic differentiation between morphotypes suggests that *Hypoplectrus* colour forms do represent more than just colour variants of a single species. However, incipient speciation cannot be assumed and these results are consistent with the possibility of an evolutionarily stable colour polymorphism.

## Supporting Information

Figure S1 **Representative images of the** ***Hypoplectrus*** **colour morphotypes at different locations included in AFLP outlier detection analysis.** N.B. Images for samples obtained in U.S. Virgin Islands not available as all individuals were released immediately after capture within this location.
(TIF)

Table S1 **Nucleotide sequences for adaptors and primers used for** ***Hypoplectrus*** **AFLP analysis.**
(DOC)

Table S2 ***F*** **st values for pair-wise comparisons of** ***Hypoplectrus*** **sympatric morphotype populations and for pair-wise same morphotype comparisons allopatric populations, based on analysis of AFLP data.**
(DOC)

Table S3 ***F*** **st values for pair-wise comparisons of** ***Hypoplectrus chlorurus*** **allopatric populations, based on analysis of AFLP data.**
(DOC)

Table S4 ***F*** **st values for pair-wise comparisons of** ***Hypoplectrus nigricans*** **allopatric populations, based on analysis of AFLP data.**
(DOC)

Table S5 ***F*** **st values for pair-wise comparisons of** ***Hypoplectrus puella*** **allopatric populations, based on analysis of AFLP data.**
(DOC)

Table S6 ***F*** **st values for pair-wise comparisons of** ***Hypoplectrus unicolor*** **allopatric populations, based on analysis of AFLP data.**
(DOC)

Table S7 **Numbers and types of polymorphic AFLP loci resulting from pair-wise comparisons of** ***Hypoplectrus*** **populations.**
(DOC)

## References

[1] Coyne JA (2007). Sympatric speciation.. Current Biology.

[2] Alexander HJ, Breden F (2004). Sexual isolation and extreme morphological divergence in the Cumaná guppy: a possible case of incipient speciation.. Journal of Evolutionary Biology.

[3] Gray SM, McKinnon JS (2007). Linking color polymorphism maintenance and speciation.. Trends in Ecology & Evolution.

[4] Pierotti MER, Seehausen O (2007). Male mating preferences pre-date the origin of a female trait polymorphism in an incipient species complex of Lake Victoria cichlids.. Journal of Evolutionary Biology.

[5] Bierne N, Bonhomme F, David P (2003). Habitat preference and the marine-speciation paradox.. Proceedings of the Royal Society B-Biological Sciences.

[6] Palumbi SR (1992). Marine speciation on a small planet.. Trends in Ecology & Evolution.

[7] Domeier ML (1994). Speciation in the serranid fish *Hypoplectrus*.. Bulletin of Marine Science.

[8] Fischer EA (1980). Speciation in the hamlets (*Hypoplectrus*: Serranidae) - a continuing enigma.. Copeia.

[9] Holt BG, Côté IM, Emerson BC (2010). Signatures of speciation? Distribution and diversity of *Hypoplectrus* (Teleostei: Serranidae) colour morphotypes.. Global Ecology and Biogeography.

[10] Holt BG, Emerson BC, Newton J, Gage MJG, Cote IM (2008). Stable isotope analysis of the *Hypoplectrus* species complex reveals no evidence for dietary niche divergence.. Marine Ecology-Progress Series.

[11] McCartney MA, Acevedo J, Heredia C, Rico C, Quenoville B (2003). Genetic mosaic in a marine species flock.. Molecular Ecology.

[12] Puebla O, Bermingham E, Guichard F (2008). Population genetic analyses of Hypoplectrus coral reef fishes provide evidence that local processes are operating during the early stages of marine adaptive radiations.. Molecular Ecology.

[13] Puebla O, Bermingham E, Guichard F, Whiteman E (2007). Colour pattern as a single trait driving speciation in *Hypoplectrus* coral reef fishes?. Proceedings of the Royal Society B-Biological Sciences.

[14] Garcia-Machado E, Monteagudo PPC, Solignac M (2004). Lack of mtDNA differentiation among hamlets (*Hypoplectrus*, Serranidae).. Marine Biology.

[15] Barreto FS, McCartney MA (2008). Extraordinary AFLP fingerprint similarity despite strong assortative mating between reef fish color morphospecies.. Evolution.

[16] Vos P, Hogers R, Bleeker M, Reijans M, Vandelee T (1995). AFLP - a new technique for DNA fingerprinting.. Nucleic Acids Research.

[17] Sambrook J, Fritsch EF, Maniatis T (1989). Molecular Cloning: a laboratory manual.

[18] Whitlock R, Hipperson H, Mannarelli M, Butlin RK, Burke T (2008). An objective, rapid and reproducible method for scoring AFLP peak-height data that minimizes genotyping error.. Molecular Ecology Resources.

[19] Vekemans X (2002). AFLP-SURV version 1.0..

[20] Beaumont MA, Nichols RA (1996). Evaluating Loci for Use in the Genetic Analysis of Population Structure.. Proceedings of the Royal Society of London Series B: Biological Sciences.

[21] Zhivotovsky LA (1999). Estimating population structure in diploids with multilocus dominant DNA markers.. Molecular Ecology.

[22] Nosil P, Egan SP, Funk DJ (2008). Heterogeneous genomic differentiation between walking-stick ecotypes: “Isolation by adaptation” and multiple roles for divergent selection.. Evolution.

[23] Foll M, Gaggiotti O (2008). A Genome-Scan Method to Identify Selected Loci Appropriate for Both Dominant and Codominant Markers: A Bayesian Perspective.. Genetics.

[24] Beaumont MA, Balding DJ (2004). Identifying adaptive genetic divergence among populations from genome scans.. Molecular Ecology.

[25] Wilding CS, Butlin RK, Grahame J (2001). Differential gene exchange between parapatric morphs of *Littorina saxatilis* detected using AFLP markers.. Journal of Evolutionary Biology.

[26] Wood HM, Grahame JW, Humphray S, Rogers J, Butlin RK (2008). Sequence differentiation in regions identified by a genome scan for local adaptation.. Molecular Ecology.

[27] Ramon ML, Lobel PS, Sorenson MD (2003). Lack of mitochondrial genetic structure in hamlets (*Hypoplectrus* spp.): recent speciation or ongoing hybridization?. Molecular Ecology.

[28] Jonsson B, Jonsson N (2001). Polymorphism and speciation in Arctic charr.. Journal of Fish Biology.

[29] Magurran AE (2001). Sexual conflict and evolution in Trinidadian guppies.. Genetica.

[30] Ryan PG, Bloomer P, Moloney CL, Grant TJ, Delport W (2007). Ecological speciation in South Atlantic island finches.. Science.

