# Supplementary figures and images for: Searching for Speciation Genes: Molecular Evidence for Selection Associated with Colour Morphotypes in the Caribbean Reef Fish Genus Hypoplectrus
Source: PLoS One. 2011 Jun 8;6(6):e20394. doi: 10.1371/journal.pone.0020394 (PMC3110725; doi:10.1371/journal.pone.0020394)

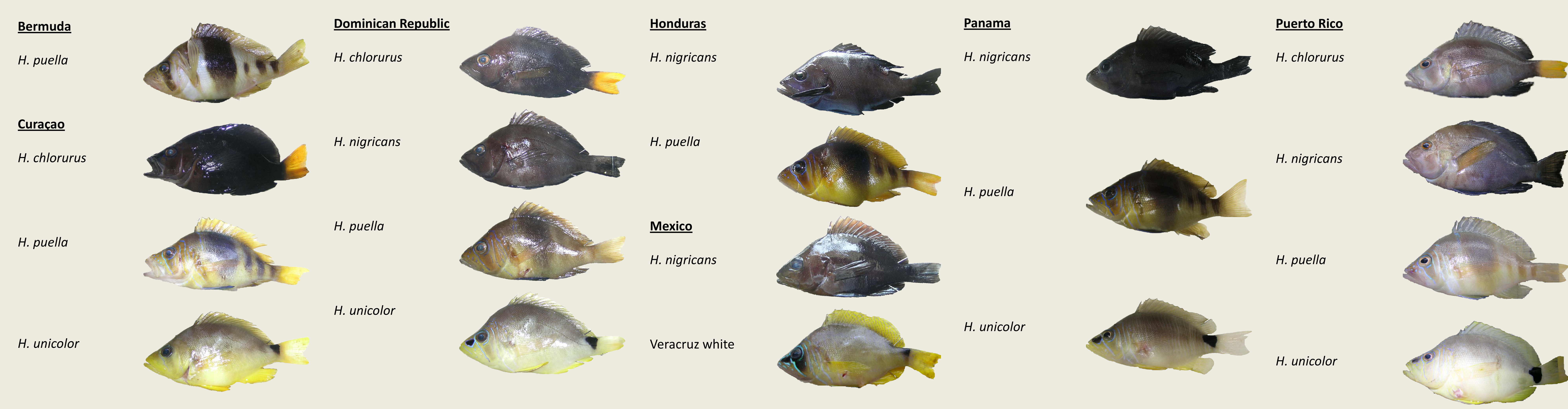

Supplement: Figure S1 — Representative images of the Hypoplectrus colour morphotypes at different locations included in AFLP outlier detection analysis. N.B. Images for samples obtained in U.S. Virgin Islands not available as all individuals were released immediately after capture within this location. (TIF) [file pone.0020394.s001.tif]
