# Supplementary material for: Searching for Speciation Genes: Molecular Evidence for Selection Associated with Colour Morphotypes in the Caribbean Reef Fish Genus Hypoplectrus
Source: PLoS One. 2011 Jun 8;6(6):e20394. doi: 10.1371/journal.pone.0020394 (PMC3110725; doi:10.1371/journal.pone.0020394)
Supplement: Table S1 — Nucleotide sequences for adaptors and primers used for Hypoplectrus AFLP analysis. (DOC) [file pone.0020394.s002.doc]

Table S1 Nucleotide sequences for adaptors and primers used for *Hypoplectrus* AFLP analysis.

|  | Sequence |
| --- | --- |
| Adaptors |  |
| EcoRI | 5’-CTCGTAGACTGCGTACC-3’  3’-CATCTGACGCATGGTTAA-5’ |
|  | 5’-AATTGGTACGCAGTCTAC-3’ |
| MseI | 5’-GACGATGAGTCCTGAG-3’  3’- TACTCAGGACTCAT-5’ |
|  | 5’-TACTCAGGACTCAT-3’ |
| Primers |  |
| Pre-Selective |  |
| Eco P | 5’-GACTGCGTACCAATTC-3’ |
| Mse P | 5’-GATGAGTCCTGAGTAA-3’ |
| Selective |  |
| Eco P: plus | AGA, ATC |
| Mse P: plus | CAC, CAG, CGT, CTC, CTT |

Pre-selective primers were used to amplify DNA specific fragments produced using restriction enzymes. Selective primers consisted of the same sequences as the pre-selective primers with three additional bases. Selective primers were used to further amplify specific subsets of the pre-selective amplification for analysis.
