# Supplementary material for: Searching for Speciation Genes: Molecular Evidence for Selection Associated with Colour Morphotypes in the Caribbean Reef Fish Genus Hypoplectrus
Source: PLoS One. 2011 Jun 8;6(6):e20394. doi: 10.1371/journal.pone.0020394 (PMC3110725; doi:10.1371/journal.pone.0020394)
Supplement: Table S2 — F st values for pair-wise comparisons of Hypoplectrus sympatric morphotype populations and for pair-wise same morphotype comparisons allopatric populations, based on analysis of AFLP data. (DOC) [file pone.0020394.s003.doc]

Table S2

| Comparison | *F*st |
| --- | --- |
| Curacao *chlorurus* vs Curacao *puella* | 0.079 |
| Curacao *chlorurus* vs Curacao *unicolor* | 0.051 |
| Curacao *puella* vs Curacao *unicolor* | 0.033 |
| Dom. Rep.  *chlorurus* vs Dom. Rep.  *nigricans* | 0.060 |
| Dom. Rep.  *chlorurus* vs Dom. Rep.  *Puella* | 0.061 |
| Dom. Rep.  *chlorurus* vs Dom. Rep.  *unicolor* | 0.058 |
| Dom. Rep.  *nigricans* vs Dom. Rep.  *Puella* | 0.021 |
| Dom. Rep.  *nigricans* vs Dom. Rep.  *unicolor* | 0.024 |
| Dom. Rep.  *puella* vs Dom. Rep.  *Unicolor* | 0.019 |
| Honduras *nigricans* vs Honduras *puella* | 0.029 |
| Mexico *nigricans* vs Mexico Veracruz white | 0.212 |
| Panama *nigricans* vs Panama *puella* | 0.052 |
| Panama *nigricans* vs Panama *unicolor* | 0.053 |
| Panama *puella* vs Panama *unicolor* | 0.058 |
| Puerto Rico *chlorurus* vs Puerto Rico *nigricans* | 0.080 |
| Puerto Rico *chlorurus* vs Puerto Rico *puella* | 0.055 |
| Puerto Rico *chlorurus* vs Puerto Rico *unicolor* | 0.038 |
| Puerto Rico *nigricans* vs Puerto Rico *puella* | 0.048 |
| Puerto Rico *nigricans* vs Puerto Rico *unicolor* | 0.061 |
| Puerto Rico *puella* vs Puerto Rico *unicolor* | 0.045 |
| U.S. Virgin Islands *chlorurus* vs U.S. Virgin Islands *puella* | 0.081 |

Values in bold are significant at the 1% level and underlined values are significant at the 5% level. Significance values are calculated using 1000 permutations and represent the % chance of finding a value as high as or higher than the empirical value. For sample sizes see Table 1 in the main article.
