# Supplementary material for: Searching for Speciation Genes: Molecular Evidence for Selection Associated with Colour Morphotypes in the Caribbean Reef Fish Genus Hypoplectrus
Source: PLoS One. 2011 Jun 8;6(6):e20394. doi: 10.1371/journal.pone.0020394 (PMC3110725; doi:10.1371/journal.pone.0020394)
Supplement: Table S3 — F st values for pair-wise comparisons of Hypoplectrus chlorurus allopatric populations, based on analysis of AFLP data. (DOC) [file pone.0020394.s004.doc]

**Table S3**

|  | Curacao | D. R. | P. R. | U.S.V.I. |
| --- | --- | --- | --- | --- |
| Curacao | - | 0.035 | 0.003 | 0.017 |
| Dom. Rep. | - | - | 0.032 | 0.008 |
| Puerto Rico | - | - | - | 0.020 |
| USVI | - | - | - | - |

Values in bold are significant at the 1% level and underlined values are significant at the 5% level. Significance values are calculated using 1000 permutations and represent the % chance of finding a value as high as or higher than the empirical value. For sample sizes see Table 1 in the main article.
