# Supplementary material for: Searching for Speciation Genes: Molecular Evidence for Selection Associated with Colour Morphotypes in the Caribbean Reef Fish Genus Hypoplectrus
Source: PLoS One. 2011 Jun 8;6(6):e20394. doi: 10.1371/journal.pone.0020394 (PMC3110725; doi:10.1371/journal.pone.0020394)
Supplement: Table S4 — F st values for pair-wise comparisons of Hypoplectrus nigricans allopatric populations, based on analysis of AFLP data. (DOC) [file pone.0020394.s005.doc]

**Table S4**

|  | Bocas | D. R. | Hon | Mex | P. R. |
| --- | --- | --- | --- | --- | --- |
| Bocas | - | **0.044** | **0.083** | **0.185** | **0.093** |
| Dom. Rep. |  | - | **0.091** | **0.175** | **0.064** |
| Honduras |  |  | - | **0.072** | **0.112** |
| Mexico |  |  |  | - | **0.223** |
| Puerto Rico |  |  |  |  | - |

Values in bold are significant at the 1% level and underlined values are significant at the 5% level. Significance values are calculated using 1000 permutations and represent the % chance of finding a value as high as or higher than the empirical value. For sample sizes see Table 1 in the main article.
