# Supplementary material for: Searching for Speciation Genes: Molecular Evidence for Selection Associated with Colour Morphotypes in the Caribbean Reef Fish Genus Hypoplectrus
Source: PLoS One. 2011 Jun 8;6(6):e20394. doi: 10.1371/journal.pone.0020394 (PMC3110725; doi:10.1371/journal.pone.0020394)
Supplement: Table S5 — F st values for pair-wise comparisons of Hypoplectrus puella allopatric populations, based on analysis of AFLP data. (DOC) [file pone.0020394.s006.doc]

***Table S5***

|  | Ber | Boc | Cur | D. R. | Hon | P. R. | U.S.V.I. |
| --- | --- | --- | --- | --- | --- | --- | --- |
| Bermuda | - | **0.055** | **0.086** | **0.043** | **0.042** | **0.065** | **0.032** |
| Bocas |  | - | 0.026 | 0.020 | **0.063** | 0.027 | **0.065** |
| Curacao |  |  | - | 0.018 | **0.109** | 0.020 | **0.096** |
| Dom. Rep. |  |  |  | - | **0.073** | 0.020 | **0.061** |
| Honduras |  |  |  |  | - | **0.085** | 0.012 |
| Puerto Rico |  |  |  |  |  | - | **0.069** |
| USVI |  |  |  |  |  |  | - |

Values in bold are significant at the 1% level and underlined values are significant at the 5% level. Significance values are calculated using 1000 permutations and represent the % chance of finding a value as high as or higher than the empirical value. For sample sizes see Table 1 in the main article.
