# Supplementary material for: Searching for Speciation Genes: Molecular Evidence for Selection Associated with Colour Morphotypes in the Caribbean Reef Fish Genus Hypoplectrus
Source: PLoS One. 2011 Jun 8;6(6):e20394. doi: 10.1371/journal.pone.0020394 (PMC3110725; doi:10.1371/journal.pone.0020394)
Supplement: Table S7 — Numbers and types of polymorphic AFLP loci resulting from pair-wise comparisons of Hypoplectrus populations. (DOC) [file pone.0020394.s008.doc]

## Table S7

| Populations | Comparision | 95% | 99% | Other | Total |
| --- | --- | --- | --- | --- | --- |
| Cc vs Cu | sd | 29 | 18 | 252 | 299 |
| Cp vs Cc | sd | 27 | 11 | 257 | 295 |
| Cp vs Cu | sd | 4 | 2 | 202 | 208 |
| Dn vs Dp | sd | 6 | 0 | 263 | 269 |
| Dn vs Du | sd | 9 | 0 | 240 | 249 |
| Dp vs Du | sd | 10 | 2 | 259 | 271 |
| Mn vs Mv | sd | 31 | 15 | 377 | 358 |
| Pn vs Pp | sd | 10 | 3 | 243 | 256 |
| PRp vs PRc | sd | 18 | 5 | 265 | 288 |
| Bp vs Cp | as | 23 | 4 | 350 | 295 |
| Bp vs Pp | as | 36 | 9 | 346 | 271 |
| Cp vs Pp | as | 8 | 3 | 211 | 208 |
| Cu vs Du | as | 7 | 3 | 233 | 249 |
| Dn vs Mn | as | 36 | 24 | 263 | 269 |
| Dp vs Pp | as | 19 | 4 | 248 | 299 |
| Hp vs Pp | as | 7 | 3 | 233 | 256 |
| Mn vs Pn | as | 27 | 7 | 270 | 271 |
| Pc vs Cc | as | 13 | 4 | 296 | 423 |
| Bp vs Pc | ad | 31 | 15 | 353 | 384 |
| Cp vs Mv | ad | 19 | 4 | 298 | 317 |
| Cu vs Dn | ad | 9 | 1 | 235 | 244 |
| Dn vs Pp | ad | 11 | 2 | 251 | 262 |
| Mn vs Pc | ad | 27 | 13 | 308 | 335 |
| Mn vs Up | ad | 27 | 13 | 308 | 335 |
| Pc vs Cu | ad | 27 | 10 | 260 | 287 |
| Pn vs Cp | ad | 16 | 5 | 189 | 205 |
| Pp vs Du | ad | 18 | 9 | 241 | 259 |

For populations B = Bermuda, C = Curacao, D = Dominican Republic, M = Mexico, P =Panama, PR = Puerto Rico, U = U.S. Virgin Islands, c = *H. chlorurus*, n = *H. nigricans*, p = *H. puella*, u = *H. unicolor* and v = Veracruz white. For comparisons sd = sympatric, different morphotypes, as = allopatric, same morphotypes and ad = allopatric, different morphotypes. 95% = loci with *F*st values above the simulated 95% quantile, 99% = loci with *F*st values above the simulated 99% quantile, Other = non outliers, Total = total number of polymorphic loci.
